# Is the “3 by 5” Initiative the Best Approach to Tackling the HIV Pandemic?

**DOI:** 10.1371/journal.pmed.0010037

**Published:** 2004-11-30

**Authors:** Jim Yong Kim, Arthur Ammann

## Abstract

Background to the debate: The World Health Organization (WHO) and its partners aim to treat 3 million people infected with HIV in poor and middle income countries with antiretroviral treatment by the end of 2005. The ambitious “3 by 5” initiative has had its supporters and its critics since its announcement in 2002.

## Abstract 2

Jim Yong Kim argues that getting 3 million HIV positive people on antiretroviral treatment by the end of 2005 is the right strategy. Arthur Ammann believes this approach is too narrow

## Jim Yong Kim's Viewpoint: 3 by 5 is a Point of Entry, Not an End in Itself

There are no sure prescriptions against great plagues like HIV. We must “learn by doing,” quickly assessing the inevitable missteps and false starts and using this information to improve outcomes. Our best information about the HIV pandemic suggests four clear principles.

First, treatment must be a core element. This does not mean treatment alone. Antiretroviral therapy (ART) is in no way more important than education, prevention of mother-to-child transmission, expanded access to testing and counseling, or other pieces of a comprehensive public health effort. But preventing mother-to-child transmission is a pyrrhic victory if, as the latest data suggest, AIDS will cause 15 million of those uninfected children to grow up as orphans [1]. Education is fruitless when the hope of finding work has disappeared because the loss of so many productive workers has led to the near collapse of industrial enterprise [2]. The dramatic benefits of ART for patients with advanced HIV disease (“the Lazarus effect”) engage the public imagination, helping to build political will on behalf of all interventions against the pandemic. And treatment can accelerate prevention by offering an incentive to get tested and know one's status, by reducing the stigma of an infection that leads to certain death, and by giving health workers credibility in devastated communities. Treatment is a point of entry, not an end in itself.

Second, time is of the essence. Every data point in the arcs of HIV transmission, morbidity, and mortality represents an exponential increase in human suffering and social and economic disruption. Countries that were able to respond early and comprehensively to HIV now have mortality rates comparable to those of Europe and North America and have cut transmission dramatically. Early interventions can provide large cost savings to the public sector and prevent devastating losses of human capital [3]. There may be risks associated with rapid scale-up—promotion of drug resistance or implementation of care delivery models that are not a perfect fit in all settings [4]. It is essential that we manage these risks through effective monitoring, evaluation, “real time” operational research, and knowledge management. However, the risks of action are minor compared with the certain failings of deferral.[Fig pmed-0010037-g001]

**Figure pmed-0010037-g001:**
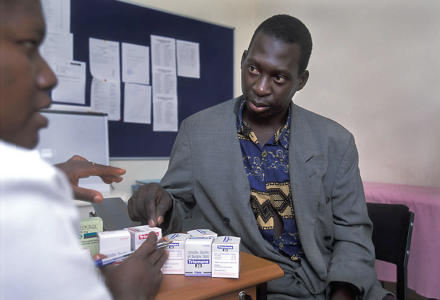
The number of patients on ART has almost doubled in the last two years (Photo: WHO/Michael Jensen)

Third, clear consensus targets are indispensable. An effective response to HIV demands the resources and attention of every region, state, and community. Coordinating these many different stakeholders requires clarity of purpose. All the great public projects of the modern era, such as the first manned moon expeditions of the 1960s, began with the establishment of a ringing collective priority. Like the 3 by 5 initiative, these projects were in themselves only surrogate endpoints. But they provided focus for the energies of their many participants. To reach the ultimate goal of universal access to ART we must plan in stages.

Fourth, the specific procedures developed to combat HIV must be codified and simplified. Because pandemics, by definition, afflict communities with a broad range of material resources and technical capacities, our methods must be suitable to the most disrupted and impoverished of them. This means effective training modules to increase the supply of health workers; it means inexpensive, rapid tests and diagnostics; and it means streamlined regimens and fixed-dose combinations that facilitate drug procurement and improve rates of adherence.

These principles are at the heart of the 3 by 5 effort. When it was announced in 2002, the gap between the damned and the saved had finally begun to narrow after sharp declines in the price of ART and the emergence of large-scale financing through the Global Fund, the World Bank, and the President's Emergency Plan for AIDS Relief. AIDS is unusual in the history of epidemics because proven, effective ways of interrupting the course of the disease have existed since shortly after its emergence, yet those methods were foreclosed to most of the world's population. The announcement of 3 by 5 drew from a widespread sense that this inequality presented unacceptable economic, political, moral, and epidemiological consequences.

Scaling up treatment is not only a possibility, but is already a reality. The number of patients on ART has nearly doubled in the last two years, mostly in countries where therapy had been limited to a privileged few [5]. High-burden countries are doing their part; the rest of the world must now share more fully in combating the pandemic. From sterile debates over prevention and treatment, the task has shifted to upgrading health systems in resource-poor settings to permit a comprehensive response to the epidemic.

Those who object to 3 by 5 must address this question: what would be the likely cost if it were never attempted? We can work exclusively to prevent the further spread of HIV, or aim to improve treatment access more slowly, but in the meanwhile high-burden countries will collapse at our feet. Or we can aim for 3 by 5 and move ourselves that much closer to the ultimate goal: preventing all unnecessary deaths from HIV.

## Arthur Ammann's Viewpoint: The Intentions Are Good, the Approach Is Wrong

There is much that is exemplary about the basic principles outlined by WHO for treatment expansion. Improving access to life-saving ART is a moral imperative and everyone agrees that this will take lots of money [6,7]. So where is the debate?

The debate lies in the strategy. Reviewing the epidemic over the past 20 years, you would have to conclude that the current international public health approach has failed and that an urgent change is required. There are three fundamental problems with the 3 by 5 approach.

First, it is very narrow, and narrow strategies for tackling HIV often fail. For example, we have known for five years that a single dose of nevirapine can help prevent perinatal HIV transmission. The intervention is simpler and cheaper than the 3 by 5 initiative—yet today less than 5% of pregnant women infected with HIV receive any ART [8,9]. WHO should instead focus on a credible overarching public health approach that is commensurate with the severity of the epidemic. Sound global public health policy requires accountability that respects the right of individuals to be protected from fatal infections. This can only be accomplished by universal offering of HIV testing, integration of HIV prevention and treatment into all health-care arenas, contact tracing, and treatment for all who require ART [8,10]. The 3 by 5 initiative fully addresses only one of these issues—treatment—and leaves HIV-exposed individuals at risk for infection and infected contacts unaware of treatment possibilities. These are irreversible missed opportunities and represent a non-accountable approach to an out-of-control epidemic.

Second, confusion about realistic costs, sources of funding, and the relation to other critical WHO initiatives abound. Initial WHO estimates of $5 billion annually for the cost of the entire 3 by 5 initiative have been revised upward to $6 billion annually [6,7,11]. It is not clear whether the costs for this program are distinct from other WHO initiatives, are incorporated into the Global Fund, will increase dramatically and continue beyond 2006, or are ultimately incorporated into national budgets. If the true cost is $6 billion annually, this is $2,000 per individual per year, which is surely not sustainable for resource-poor countries. Further, of the 40 million individuals infected with HIV worldwide, it is likely that at least 20 million will require treatment, and there will be millions more newly diagnosed patients [11]. This would conservatively place treatment costs at over $40 billion per year. Only recently has WHO acknowledged what everyone else seemed to know from the beginning—it will take huge investments in infrastructure to sustain ART delivery [7,12,13,14].

Third, the 3 by 5 initiative is a “top down” unsustainable approach that, without a high level of government investment, fosters dependence on international aid. Countries that have taken ownership of their HIV programs have often been the most successful in tackling the epidemic [8,14,15]. Brazil has mounted an effective response to the HIV epidemic whereas South Africa has not. A key difference between these countries is that 84% of Brazil's AIDS programs are funded from domestic sources compared to 0.4% in South Africa [6]. Furthermore, there are thousands of nongovernmental organizations, clinics, and hospitals already treating patients with HIV and a “top down” approach doesn't work for these organizations. Many are up and running and require only minimal training to move ART distribution forward. The last thing they need is more internationally imposed hurdles that ensure sequestration of ART in costly and inefficient bureaucracies.

The HIV epidemic is the worst pandemic in history. Why, then, is the international public health response so disparate from public health responses to other life-threatening infectious diseases? The 3 by 5 initiative, with no requirement for contact tracing, does not ensure the right of uninfected individuals to be protected or of infected contacts to gain access to treatment. It is time to acknowledge that the severity of the epidemic requires universal offering of HIV testing and counseling, contact tracing, and integration of sound public health prevention and treatment principles into all health-care delivery systems [8,10].[Fig pmed-0010037-g002]

**Figure pmed-0010037-g002:**
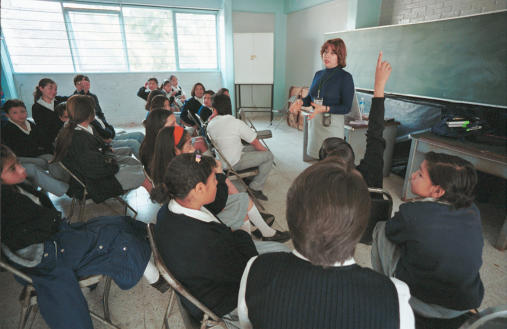
Prevention efforts—including education—must be at the heart of tackling HIV (Photo: Rick Maiman, on behalf of the David and Lucile Packard Foundation)

Thousands of existing and new teams of health-care workers should be trained in voluntary testing and counseling, and in treatment, using streamlined training courses. Mobile teams could be used to reach rural areas where as many as 50% of infected individuals may live. Cumbersome “top down” training and certification, as is currently planned, will only delay the very therapy that WHO seeks to make more available [8,14,15].

It is a paradox that HIV is one of the few diseases that is deemed to require exceptional international and national bodies overseeing access to medicines. The success of ART in developed countries was a result of making ART directly available to those who treat patients, not to governments. Widespread and timely access can only occur when health workers are able to provide ART without repressive procedures. We can distribute drugs more freely for other diseases—why not HIV?

## Kim's Response to Ammann's Viewpoint

I agree with Arthur Ammann that the response of the international community to the HIV epidemic over the last 20 years has been grossly inadequate. I also agree that a more effective strategy must pay close attention to mother-to-child transmission, provide “opt out” testing and counseling integrated into key health-care settings, implement effective partner referral strategies, and train many thousands of health-care workers. These are stated WHO priorities—integral to the 3 by 5 initiative—as any attempt to investigate our position would reveal [16,17].

Dr. Ammann's other charges are also puzzling. Our latest cost projections (which he cites) in fact provide a two-year, not annual, range of $5.1 to $5.9 billion, depending on the price of drugs [7]. The accusation of a “top down” approach seems misplaced. WHO's primary contact is indeed with ministries of health, given our constitutionally mandated obligation to provide technical assistance for 192 WHO member states. But I strongly dispute that this relationship somehow prejudices the 3 by 5 agenda against nongovernmental organizations or local providers. WHO is far from the only partner in this struggle, and we actively promote broad-based collaborations within the nonprofit sector, the private sector, among traditional healers, and within civil society [18].

Finally, I must point out a contradiction in Dr. Ammann's arguments. On the one hand, he urges high-burden countries to take ownership of their HIV programs and finance these themselves. On the other, he acknowledges that if the “true cost [of ART] is $6 billion annually, this is $2,000 per individual per year, which is surely not sustainable for resource-poor countries.” The crucial question is whether $6 billion annually is “sustainable” for the world community as a whole. In facing the worst health disaster in several centuries, we simply cannot wait for the poorest countries to self-finance HIV treatment—or else we will be guilty of standing idly by as millions die and societies collapse.

## Ammann's Response to Kim's Viewpoint

There are time-tested prescriptions for halting epidemics, and HIV should be no exception. But “HIV exceptionalism” persists [19]. Current public health efforts fail to insist on universal HIV testing and contact tracing, thereby limiting treatment and prevention opportunities and contributing to “feminization” of the epidemic (more and more women getting infected at an earlier age) [20]. Prevention and treatment are inextricably linked. The 3 by 5 initiative should meet the high standards—individual and organizational accountability, justice, and public good—of other public health approaches.

Prevention of perinatal HIV transmission cannot be called a victory—not even a pyrrhic one—since less than 5% of pregnant women infected with HIV receive any ART. Further, in most perinatal HIV prevention programs sexual partners are generally not identified, treatment has only recently been offered, and HIV testing has not been universally implemented. Narrowly focused, top-down programs such as the 3 by 5 initiative face similar outcomes when they adopt yet another partial approach that is not fully integrated into countrywide general health care.

The 3 by 5 initiative suggests that treatment is the major incentive for HIV testing. But getting tested has two incentives—treatment and prevention. Knowing one's HIV status is a means of saving lives, an incentive that should motivate every individual to be tested even when treatment is not available. We will destine ourselves to an ever-escalating epidemic if we delay universal testing until treatment is available.

Both Jim Yong Kim and I want to treat 3 million individuals infected with HIV and more, and we agree that time is of the essence. But why take another decade to learn what we already know from epidemics such as tuberculosis? Lateral, comprehensive approaches that equip health-care workers with testing and the required drugs work best [21,22]. Procedures to combat HIV have already been simplified by such workers in resource-poor areas—so get the tests and the drugs to them and they will do the treating.

## Abbreviations

ART: antiretroviral therapy
WHO: World Health Organization
